# *N*-Phenyl­succinamic acid

**DOI:** 10.1107/S160053681005364X

**Published:** 2011-01-08

**Authors:** B. Thimme Gowda, Sabine Foro, B. S. Saraswathi, Hartmut Fuess

**Affiliations:** a Department of Chemistry, Mangalore University, Mangalagangotri 574 199, Mangalore, India; b Institute of Materials Science, Darmstadt University of Technology, Petersenstrasse 23, D-64287 Darmstadt, Germany

## Abstract

In the crystal structure of the title compound, C_10_H_11_NO_3_, the conformations of N—H and C=O bonds in the amide segment are *anti* to each other. Further, the conformations of the amide O atom and the carbonyl O atom of the acid segment are *anti* to each other and to the adjacent –CH_2_ groups. The C=O and O—H bonds of the acid group are in *syn* positions with respect to each other. In the crystal, the mol­ecules are packed into infinite chains along the *a* axis through inter­molecular N—H⋯O and O—H⋯O hydrogen bonds.

## Related literature

For our studies of the effect of substituents on the structures of anilides, see: Gowda *et al.* (2009 ▶, 2010**a* ▶,b* ▶). For modes of inter­linking carb­oxy­lic acids by hydrogen bonds, see: Leiserowitz (1976 ▶). For the packing of mol­ecules involving dimeric hydrogen-bonded association of each carboxyl group with a centrosymmetrically related neighbor, see: Jagannathan *et al.* (1994 ▶).
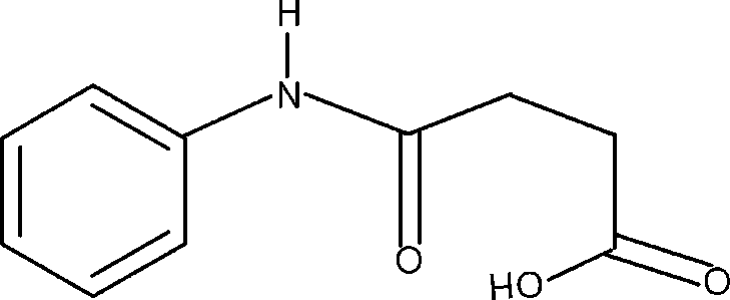


## Experimental

### 

#### Crystal data

C_10_H_11_NO_3_
*M* *_r_* = 193.20
Monoclinic, 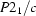

*a* = 4.986 (1) Å
*b* = 25.108 (4) Å
*c* = 7.895 (2) Å
β = 103.18 (2)°
*V* = 962.3 (3) Å^3^
*Z* = 4
Mo *K*α radiation
μ = 0.10 mm^−1^
*T* = 293 K
0.44 × 0.14 × 0.14 mm

#### Data collection

Oxford Diffraction Xcalibur diffractometer with a Sapphire CCD detector
Absorption correction: multi-scan (*CrysAlis RED*; Oxford Diffraction, 2009 ▶) *T*_min_ = 0.958, *T*_max_ = 0.986
3269 measured reflections
1791 independent reflections
1033 reflections with *I* > 2σ(*I*)
*R*_int_ = 0.022

#### Refinement

*R*[*F* ^2^ > 2σ(*F* ^2^)] = 0.053
*wR*(*F* ^2^) = 0.135
*S* = 0.99
1791 reflections
133 parameters
2 restraints
H atoms treated by a mixture of independent and constrained refinement
Δρ_max_ = 0.15 e Å^−3^
Δρ_min_ = −0.13 e Å^−3^

### 

Data collection: *CrysAlis CCD* (Oxford Diffraction, 2009 ▶); cell refinement: *CrysAlis RED* (Oxford Diffraction, 2009 ▶); data reduction: *CrysAlis RED*; program(s) used to solve structure: *SHELXS97* (Sheldrick, 2008 ▶); program(s) used to refine structure: *SHELXL97* (Sheldrick, 2008 ▶); molecular graphics: *PLATON* (Spek, 2009 ▶); software used to prepare material for publication: *SHELXL97*.

## Supplementary Material

Crystal structure: contains datablocks I, global. DOI: 10.1107/S160053681005364X/bq2265sup1.cif

Structure factors: contains datablocks I. DOI: 10.1107/S160053681005364X/bq2265Isup2.hkl

Additional supplementary materials:  crystallographic information; 3D view; checkCIF report

## Figures and Tables

**Table 1:** Hydrogen-bond geometry (Å, °)

*D*—H⋯*A*	*D*—H	H⋯*A*	*D*⋯*A*	*D*—H⋯*A*
N1—H1*N*⋯O1^i^	0.85 (2)	2.22 (2)	3.041 (3)	161 (2)
O3—H3*O*⋯O2^ii^	0.88 (2)	1.80 (2)	2.671 (2)	177 (3)

Symmetry codes: (i) 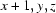; (ii) 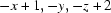.

**Fig. 1.**
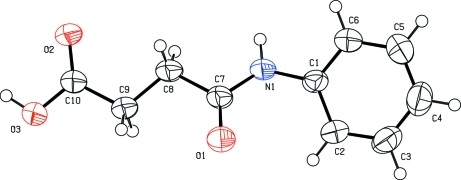
Molecular structure of the title compound, showing the atom labeling scheme. The displacement ellipsoids are drawn at the 50% probability level.

**Fig. 2.**
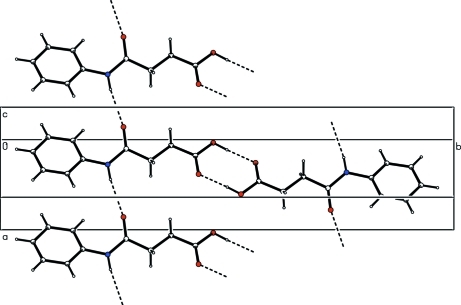
Molecular packing of the title compound with hydrogen bonding shown as dashed lines.

## supplementary crystallographic information

### Comment

As a part of studying the effect of ring and side chain substitutions on the crystal structures of anilides (Gowda *et al.*, 2009; 2010**a*,b*), the crystal structure of *N*-(phenyl)succinamic acid (I) has been determined. The conformations of N—H and C=O bonds in the amide segment are *anti* to each other. The conformation of the amide oxygen and the carbonyl oxygen of the acid segment are also *anti* to each other, similar to that observed in *N*-(2-chlorophenyl)succinamic acid (II) (Gowda *et al.*, 2009) and *N*-(2-methylphenyl)succinamic acid (III)(Gowda *et al.*, 2010*b*), but contrary to the *syn* conformation observed in *N*-(3-methylphenyl)succinamic acid (IV) and *N*-(3-chlorophenyl)succinamic acid (V) (Gowda *et al.*, 2010*a*). Further, the conformation of both the C=O bonds are *anti* to the H atoms of their adjacent –CH_2_ groups (Fig. 1) and the C=O and O—H bonds of the acid group are in *syn* position to each other, similar to that observed in (II), (III), (IV) and (V).

The N—H···O and O—H···O intermolecular hydrogen bonds pack the molecules into infinite chains in the structure (Table 1, Fig.2).

The modes of interlinking carboxylic acids by hydrogen bonds is described elsewhere (Leiserowitz, 1976). The packing of molecules involving dimeric hydrogen bonded association of each carboxyl group with a centrosymmetrically related neighbor has also been observed (Jagannathan *et al.*, 1994).

### Experimental

The solution of succinic anhydride (0.01 mole) in toluene (25 ml) was treated dropwise with the solution of aniline (0.01 mole) also in toluene (20 ml) with constant stirring. The resulting mixture was stirred for about one h and set aside for an additional hour at room temperature for completion of the reaction. The mixture was then treated with dilute hydrochloric acid to remove the unreacted aniline. The resultant solid *N*-(phenyl)succinamic acid was filtered under suction and washed thoroughly with water to remove the unreacted succinic anhydride and succinic acid. It was recrystallized to constant melting point from ethanol. The purity of the compound was checked by elemental analysis and characterized by its infrared and NMR spectra.

Prism like colorless single crystals used in x-ray diffraction studies were grown in ethanolic solution by slow evaporation at room temperature.

### Refinement

The H atoms of the NH and OH group were located in a difference map and later restrained to the distance N—H = 0.86 (2) Å and O—H = 0.82 (2) Å. The other H atoms were positioned with idealized geometry using a riding model with C—H = 0.93–0.97 Å. All H atoms were refined with isotropic displacement parameters (set to 1.2 times of the *U*_eq_ of the parent atom).

### Crystal data

**Table:**

C_10_H_11_NO_3_	*F*(000) = 408
*M**_r_* = 193.20	*D*_x_ = 1.333 Mg m^−^^3^
Monoclinic, *P*2_1_/*c*	Mo *K*α radiation, λ = 0.71073 Å
Hall symbol: -P 2ybc	Cell parameters from 702 reflections
*a* = 4.986 (1) Å	θ = 3.1–27.7°
*b* = 25.108 (4) Å	µ = 0.10 mm^−^^1^
*c* = 7.895 (2) Å	*T* = 293 K
β = 103.18 (2)°	Prism, colorless
*V* = 962.3 (3) Å^3^	0.44 × 0.14 × 0.14 mm
*Z* = 4	

### Data collection

**Table:**

Oxford Diffraction Xcalibur diffractometer with a Sapphire CCD detector	1791 independent reflections
Radiation source: fine-focus sealed tube	1033 reflections with *I* > 2σ(*I*)
graphite	*R*_int_ = 0.022
Rotation method data acquisition using ω scans	θ_max_ = 25.7°, θ_min_ = 3.1°
Absorption correction: multi-scan (*CrysAlis RED*; Oxford Diffraction, 2009)	*h* = −5→6
*T*_min_ = 0.958, *T*_max_ = 0.986	*k* = −30→22
3269 measured reflections	*l* = −7→9

### Refinement

**Table:**

Refinement on *F*^2^	Primary atom site location: structure-invariant direct methods
Least-squares matrix: full	Secondary atom site location: difference Fourier map
*R*[*F*^2^ > 2σ(*F*^2^)] = 0.053	Hydrogen site location: inferred from neighbouring sites
*wR*(*F*^2^) = 0.135	H atoms treated by a mixture of independent and constrained refinement
*S* = 0.99	*w* = 1/[σ^2^(*F*_o_^2^) + (0.0703*P*)^2^] where *P* = (*F*_o_^2^ + 2*F*_c_^2^)/3
1791 reflections	(Δ/σ)_max_ < 0.001
133 parameters	Δρ_max_ = 0.15 e Å^−^^3^
2 restraints	Δρ_min_ = −0.13 e Å^−^^3^

### Special details

**Table:**

Experimental. CrysAlis RED (Oxford Diffraction, 2009) Empirical absorption correction using spherical harmonics, implemented in SCALE3 ABSPACK scaling algorithm.
Geometry. All e.s.d.'s (except the e.s.d. in the dihedral angle between two l.s. planes) are estimated using the full covariance matrix. The cell e.s.d.'s are taken into account individually in the estimation of e.s.d.'s in distances, angles and torsion angles; correlations between e.s.d.'s in cell parameters are only used when they are defined by crystal symmetry. An approximate (isotropic) treatment of cell e.s.d.'s is used for estimating e.s.d.'s involving l.s. planes.
Refinement. Refinement of *F*^2^ against ALL reflections. The weighted *R*-factor *wR* and goodness of fit *S* are based on *F*^2^, conventional *R*-factors *R* are based on *F*, with *F* set to zero for negative *F*^2^. The threshold expression of *F*^2^ > σ(*F*^2^) is used only for calculating *R*-factors(gt) *etc*. and is not relevant to the choice of reflections for refinement. *R*-factors based on *F*^2^ are statistically about twice as large as those based on *F*, and *R*- factors based on ALL data will be even larger.

### Fractional atomic coordinates and isotropic or equivalent isotropic displacement parameters (Å^2^)

**Table:**

	*x*	*y*	*z*	*U*_iso_*/*U*_eq_	
C1	0.3834 (4)	0.31826 (10)	0.9635 (3)	0.0421 (6)	
C2	0.1411 (5)	0.33972 (11)	0.8656 (3)	0.0543 (7)	
H2	0.0058	0.3177	0.8010	0.065*	
C3	0.1030 (6)	0.39415 (12)	0.8651 (4)	0.0649 (8)	
H3	−0.0589	0.4086	0.7989	0.078*	
C4	0.2995 (6)	0.42738 (12)	0.9604 (4)	0.0702 (9)	
H4	0.2712	0.4640	0.9589	0.084*	
C5	0.5378 (6)	0.40589 (12)	1.0577 (4)	0.0697 (9)	
H5	0.6720	0.4280	1.1229	0.084*	
C6	0.5793 (5)	0.35204 (11)	1.0595 (3)	0.0562 (7)	
H6	0.7418	0.3379	1.1263	0.067*	
C7	0.2674 (5)	0.22230 (10)	0.9613 (3)	0.0448 (6)	
C8	0.4033 (4)	0.16836 (10)	0.9852 (3)	0.0484 (7)	
H8A	0.5575	0.1683	0.9293	0.058*	
H8B	0.4757	0.1622	1.1085	0.058*	
C9	0.2123 (4)	0.12352 (9)	0.9121 (3)	0.0504 (7)	
H9A	0.0499	0.1255	0.9601	0.060*	
H9B	0.1531	0.1280	0.7871	0.060*	
C10	0.3391 (5)	0.06985 (10)	0.9496 (3)	0.0491 (7)	
N1	0.4434 (4)	0.26322 (8)	0.9654 (3)	0.0483 (6)	
H1N	0.616 (3)	0.2572 (10)	0.983 (3)	0.058*	
O1	0.0199 (3)	0.22839 (7)	0.9463 (3)	0.0677 (6)	
O2	0.5739 (3)	0.06282 (7)	1.0309 (3)	0.0701 (6)	
O3	0.1737 (4)	0.03061 (7)	0.8868 (3)	0.0728 (7)	
H3O	0.262 (5)	0.0004 (8)	0.913 (4)	0.087*	

### Atomic displacement parameters (Å^2^)

**Table:**

	*U*^11^	*U*^22^	*U*^33^	*U*^12^	*U*^13^	*U*^23^
C1	0.0303 (11)	0.0465 (16)	0.0499 (15)	0.0021 (11)	0.0102 (10)	0.0057 (12)
C2	0.0405 (14)	0.0548 (18)	0.0616 (17)	0.0040 (12)	−0.0005 (12)	0.0061 (14)
C3	0.0508 (16)	0.063 (2)	0.077 (2)	0.0145 (15)	0.0075 (14)	0.0158 (17)
C4	0.070 (2)	0.0465 (18)	0.097 (2)	0.0067 (16)	0.0269 (17)	0.0088 (17)
C5	0.0595 (19)	0.052 (2)	0.094 (2)	−0.0094 (15)	0.0099 (16)	−0.0023 (17)
C6	0.0377 (13)	0.0542 (18)	0.072 (2)	−0.0007 (13)	0.0018 (12)	0.0006 (14)
C7	0.0301 (13)	0.0461 (16)	0.0578 (16)	0.0006 (11)	0.0089 (10)	−0.0025 (12)
C8	0.0288 (12)	0.0505 (16)	0.0640 (17)	0.0025 (11)	0.0064 (11)	−0.0015 (13)
C9	0.0330 (12)	0.0470 (16)	0.0667 (17)	0.0044 (11)	0.0020 (11)	0.0003 (13)
C10	0.0344 (14)	0.0473 (16)	0.0619 (17)	−0.0001 (12)	0.0037 (12)	0.0007 (13)
N1	0.0241 (10)	0.0463 (14)	0.0721 (15)	0.0022 (10)	0.0057 (10)	0.0010 (11)
O1	0.0252 (9)	0.0548 (12)	0.1239 (17)	0.0050 (8)	0.0184 (9)	0.0050 (11)
O2	0.0411 (10)	0.0472 (11)	0.1055 (16)	0.0068 (8)	−0.0175 (10)	−0.0023 (10)
O3	0.0426 (10)	0.0436 (11)	0.1161 (17)	−0.0035 (9)	−0.0156 (10)	0.0047 (11)

### Geometric parameters (Å, °)

**Table:**

C1—C6	1.382 (3)	C7—N1	1.347 (3)
C1—C2	1.386 (3)	C7—C8	1.507 (3)
C1—N1	1.413 (3)	C8—C9	1.503 (3)
C2—C3	1.380 (4)	C8—H8A	0.9700
C2—H2	0.9300	C8—H8B	0.9700
C3—C4	1.373 (4)	C9—C10	1.489 (3)
C3—H3	0.9300	C9—H9A	0.9700
C4—C5	1.370 (4)	C9—H9B	0.9700
C4—H4	0.9300	C10—O2	1.213 (3)
C5—C6	1.367 (4)	C10—O3	1.308 (3)
C5—H5	0.9300	N1—H1N	0.852 (16)
C6—H6	0.9300	O3—H3O	0.877 (17)
C7—O1	1.222 (3)		
			
C6—C1—C2	119.0 (2)	N1—C7—C8	114.25 (19)
C6—C1—N1	118.2 (2)	C9—C8—C7	113.43 (18)
C2—C1—N1	122.8 (2)	C9—C8—H8A	108.9
C3—C2—C1	119.2 (2)	C7—C8—H8A	108.9
C3—C2—H2	120.4	C9—C8—H8B	108.9
C1—C2—H2	120.4	C7—C8—H8B	108.9
C4—C3—C2	121.3 (3)	H8A—C8—H8B	107.7
C4—C3—H3	119.3	C10—C9—C8	113.48 (19)
C2—C3—H3	119.3	C10—C9—H9A	108.9
C5—C4—C3	119.1 (3)	C8—C9—H9A	108.9
C5—C4—H4	120.4	C10—C9—H9B	108.9
C3—C4—H4	120.4	C8—C9—H9B	108.9
C6—C5—C4	120.4 (3)	H9A—C9—H9B	107.7
C6—C5—H5	119.8	O2—C10—O3	122.7 (2)
C4—C5—H5	119.8	O2—C10—C9	123.5 (2)
C5—C6—C1	121.0 (2)	O3—C10—C9	113.83 (19)
C5—C6—H6	119.5	C7—N1—C1	127.64 (19)
C1—C6—H6	119.5	C7—N1—H1N	119.7 (18)
O1—C7—N1	123.0 (2)	C1—N1—H1N	112.3 (18)
O1—C7—C8	122.7 (2)	C10—O3—H3O	108.8 (19)
			
C6—C1—C2—C3	0.7 (4)	N1—C7—C8—C9	−157.0 (2)
N1—C1—C2—C3	−177.3 (2)	C7—C8—C9—C10	−174.8 (2)
C1—C2—C3—C4	−0.4 (5)	C8—C9—C10—O2	−0.1 (4)
C2—C3—C4—C5	0.0 (5)	C8—C9—C10—O3	179.8 (2)
C3—C4—C5—C6	0.1 (5)	O1—C7—N1—C1	3.7 (4)
C4—C5—C6—C1	0.1 (4)	C8—C7—N1—C1	−173.5 (2)
C2—C1—C6—C5	−0.5 (4)	C6—C1—N1—C7	144.5 (3)
N1—C1—C6—C5	177.5 (2)	C2—C1—N1—C7	−37.5 (4)
O1—C7—C8—C9	25.8 (4)		

### Hydrogen-bond geometry (Å, °)

**Table:**

*D*—H···*A*	*D*—H	H···*A*	*D*···*A*	*D*—H···*A*
N1—H1N···O1^i^	0.85 (2)	2.22 (2)	3.041 (3)	161 (2)
O3—H3O···O2^ii^	0.88 (2)	1.80 (2)	2.671 (2)	177 (3)

Symmetry codes: (i) *x*+1, *y*, *z*; (ii) −*x*+1, −*y*, −*z*+2.
